# Bayesian evaluation of behavior change interventions: a brief introduction and a practical example

**DOI:** 10.1080/21642850.2018.1428102

**Published:** 2018-04-11

**Authors:** Matti T. J. Heino, Matti Vuorre, Nelli Hankonen

**Affiliations:** a Department of Social Research, University of Helsinki, Helsinki, Finland; b Faculty of Social Sciences, University of Tampere, Tampere, Finland; c Department of Psychology, Columbia University, New York, NY, USA

**Keywords:** Bayes, Bayesian estimation, health behavior change, intervention evaluation, tutorial

## Abstract

**Introduction:**

Evaluating effects of behavior change interventions is a central interest in health psychology and behavioral medicine. Researchers in these fields routinely use frequentist statistical methods to evaluate the extent to which these interventions impact behavior and the hypothesized mediating processes in the population. However, calls to move beyond the exclusive use of frequentist reasoning are now widespread in psychology and allied fields. We suggest adding Bayesian statistical methods to the researcher’s toolbox of statistical methods.

**Objectives:**

We first present the basic principles of the Bayesian approach to statistics and why they are useful for researchers in health psychology. We then provide a practical example on how to evaluate intervention effects using Bayesian methods, with a focus on Bayesian hierarchical modeling. We provide the necessary materials for introductory-level readers to follow the tutorial.

**Conclusion:**

Bayesian analytical methods are now available to researchers through easy-to-use software packages, and we recommend using them to evaluate the effectiveness of interventions for their conceptual and practical benefits.

## Introduction

Bayesian inference, after being conceived by the clergyman Thomas Bayes and astronomer-mathematician Pierre-Simon Laplace in the 1700s, spent two centuries in relative obscurity before surfacing again in the mid-1900s, with the rise of modern computing (McGrayne, 2011). Since then, much ink has been spilled over discussions about the validity and relative benefits of different statistical approaches (Efron, 2013). It may then come as a surprise that many statisticians now consider these debates outdated: ‘We have all, or nearly all, moved past these old debates, yet our textbook explanations have not caught up with the eclecticism of statistical practice’ (Kass, 2011). Further, there has long been a broad agreement that consumers of applied statistics need to move beyond null hypothesis significance testing as it is traditionally conducted (Benjamin et al., 2017; Cumming, 2014; Gigerenzer, Krauss, & Vitouch, 2004; Kruschke, 2010; Lakens et al., 2017; McShane, Gal, Gelman, Robert, & Tackett, 2017; Nickerson, 2000).

Accordingly, Bayesian statistical methods have recently experienced a surge in popularity in psychology and other disciplines (Andrews & Baguley, 2013; van de Schoot, Winter, Ryan, Zondervan-Zwijnenburg, & Depaoli, 2017), reaching mainstream health psychology recently (Beard & West, 2017; Depaoli, Rus, & Clifton, 2017). The Bayesian approach to inference is especially attractive in the context of health psychology for several reasons. For example, Bayesian methods perform well with small sample sizes (van de Schoot, Broere, Perryck, Zondervan-Zwijnenburg, & Van Loey, 2015), which is of importance to health psychologists in many areas. In addition, Bayesian methods perform well with complex statistical models such as multilevel structural equation modeling (Depaoli & Clifton, 2015; Vuorre & Bolger, 2017) and growth mixture modeling (Depaoli, 2013) – but also simpler ones examining differences between two groups (Kruschke, 2013). Powerful robust methods are now emerging for analyzing heterogeneous data (Williams & Martin, 2017). Also, Bayesian methods allow for the researcher to incorporate prior information regarding the research topic in evaluating the data, which allows for improvements in out-of-sample prediction.

In this tutorial, we present an introductory-level overview on the Bayesian approach to statistical inference and a practical tutorial on applying Bayesian methods to analyzing effects of behavior change interventions that use an experimental design. Because our aim is to present a hands-on introductory tutorial for beginners, wherever applicable we refer the reader to further resources for a more in-depth understanding. In addition to the conceptual part, researchers who mainly act as reviewers and might not need to conduct Bayesian analyses themselves may find the annotated reading list by Etz, Gronau, Dablander, Edelsbrunner, and Baribault (2017) useful.

## Evaluating interventions as a key research interest

Evaluating effects and processes of health behavior change interventions is an increasingly studied topic in the field of health psychology and behavioral medicine. Intervention studies can help identify the most effective solutions to promote health and prevent disease in specific populations and target behaviors and provide a useful platform to test and refine theories of health behavior change (Rothman, 2004). Indeed, the U.K. Medical Research Council guidance on a process evaluation of complex interventions (Moore et al., 2015), as well as the WIDER consensus statement (Abraham, Johnson, de Bruin, & Luszczynska, 2014), call for increased attention to the postulated processes underpinning behavior change. To draw reliable and appropriate conclusions (for both practice and theory), we need not only a good theory, a rigorous study design and high-quality data collection procedures, but also a sound analytical approach to understand the data.

Complex health behavior intervention studies are often designed to a specific population, usually require a long time to plan carefully, and are arguably even less often directly replicated than is the case in psychology in general (Makel, Plucker, & Hegarty, 2012). Due to a large amount of resources needed for data collection in the field rather than in the laboratory setting, it is often not possible to gather additional participants when attrition reaches surprisingly high levels, or when the recruitment plan turns out overly optimistic. On the other hand, recruitment may be a success, but for the quantitative process evaluation, the complexity of the intervention requires a more complex statistical model for assessing its mechanisms, than what the trial was powered for. These are just some examples of situations where Bayes can help.

Hence, an intervention researcher may use the Bayesian methods in various phases of an intervention study: In the definitive randomized controlled trial (RCT), a key interest lies in the evaluation of the effectiveness of the intervention in changing the primary outcome(s). Additionally, a Bayesian approach could be taken to evaluate the psychosocial or other *processes* explaining the causal mechanism behind the intervention effect on the outcome (or a lack thereof).

Furthermore, Bayesian evaluation could also be used in the earlier phase of feasibility testing and piloting, and optimization of the intervention prior to full trial: To make sure that work is not thrown to waste because of unwarranted assumptions, many guidelines recommend that measures and delivery of an intervention be tested in small scale before embarking in a definitive RCT to evaluate its effectiveness (e.g. Craig et al., 2008). In such studies, one possible use of Bayesian inference could be a preliminary investigation of intervention effects on its hypothesized impact mechanisms via determinants (e.g. attitudes, motivation) or even outcomes.

### Example dataset: intervening on physical activity motivation

This tutorial uses a dataset from a recent study examining the feasibility and acceptability of the ‘Let’s Move It’ intervention and planned trial procedures (Hankonen et al., 2017), prior to a definitive effectiveness trial. The aim of this multilevel, school-based intervention was to increase physical activity (PA) and decrease sedentary behavior among older adolescents (Hankonen et al., 2016). The intervention included several components, e.g. six weekly group sessions, delivered in the context of a health education course, to increase motivation and self-regulation skills to promote leisure-time PA, poster campaign, teacher training for reducing excessive sitting in classrooms, etc. The focus of this tutorial is on the PA change and the student dataset (*n* = 43). Four student groups, randomized into control and intervention arms, were measured at baseline (T1) and after the intensive intervention at approximately six weeks (T2).

The program theory of this complex intervention hypothesized several mechanisms of action. One of the key hypothesized mechanisms leading to increased PA, based on the self-determination theory (Ryan & Deci, 2000), are the positive changes in the quality of motivation, i.e. internalization of motivational regulation. The intervention attempts to deliver autonomy supportive and motivational interaction, prompting participants to find personally meaningful and intrinsically motivated reasons to engage in PA, as opposed to controlled motivation, e.g. engaging in PA for extrinsic reasons such as avoiding external punishment or feelings of guilt or shame.

As is often the case in such feasibility studies, this sample size is relatively small, as their primary objectives include investigations of acceptability to participants and/or providers, and feasibility of the study design and intervention. (‘A feasibility study asks whether something can be done, should we proceed with it, and if so, how’; Eldridge et al., 2016). Hence, the study did not aim to reliably detect hypothesized changes in outcomes. But does this mean that the collected data are uninformative regarding those changes? Traditional null hypothesis significance testing suggests not much has been learned, but a Bayesian estimation perspective can provide a richer perspective to the investigation.

In our case, it was assumed that a change in the determinant should be (possibly much) higher than the expected subsequent change in the outcome; hence, it might be possible to extract useful information from the study even with the small sample available. But we do not know this before we examine the data. Such information in similar pilot studies could then be used to inform and/or modify a definitive RCT that is set to follow.

For our demonstration purposes, the case at hand is now used to investigate the intervention’s effects on determinants of PA change, or on the other hand, the plausibility of the intervention causing counterproductive effects. Specifically, the research question is: ‘To what extent does the intervention affect autonomous motivation?’. We now turn to introducing the foundations of Bayesian inference, and then show how to use them to answer this research question.

We will keep the discussion about the intricacies of Bayes on a general level and focus on practicalities in this tutorial. We encourage the reader to look into ongoing discussions about the differences between objective, subjective and falsificationist Bayes, and how the standard model of Bayesian inference as subjective and inductive is very much debatable (Gelman, 2011; Gelman & Hennig, 2017; Gelman & Shalizi, 2013).

## Bayesian inference

In the example case, we are interested in modeling the change of autonomous motivation over time, and how that change differs between the intervention and control groups. Conventionally, one would estimate the effect and calculate the *p*-value^1^: How probable would this – or more extreme – data be in the long run, if the effect was zero (i.e. null hypothesis was true).

Instead of considering the long-term implications of the observed or more extreme data given the null hypothesis, Bayesians consider the data fixed, and inspect processes that could describe such data. These processes are represented as assumed models, which have certain settings, or parameters^2^. Parameter values are then evaluated based on their capacity to generate data that matches the observed data.

This brings us to a major difference between the Bayesian and frequentist approaches: the meaning of probability. Frequentists consider probability as long-run frequency from a very long (or infinite) sequence of repetitions. For Bayesians, the probability is a measure of uncertainty associated with unknown quantities, such as the parameters in a model.

What a Bayesian seeks is the probability of a parameter, given the data – written as p(parameter∣data). This value is found by taking advantage of a certain property of conditional probability:

p(B∣A)×p(A)=p(A∣B)×p(B).

We can substitute A and B with parameter and data;

p(parameter∣data)×p(data)=p(data∣parameter)×p(parameter).

Dividing both sides by the probability of data, we get:

$$p(\text{parameter}\;|\;\text{data})=\frac{p(\text{data}\;|\;\text{parameter})\times p(\text{parameter})}{p(\text{data})}.$$

The expression is essentially what is known as the *Bayes’ theorem*, which is often recognized as:

$$\text{posterior}=\frac{\text{likelihood}\times \text{prior}}{\text{average likelihood}}.$$

We can also think of the posterior being the likelihood multiplied by the prior and a normalizing constant. So, one way to put the above is to say that ‘the posterior is proportional to the likelihood multiplied by the prior’. These terms will be presented next.

### The three components of Bayes

Bayesian inference deals with information in terms of *probability distributions*. Uncertainty in e.g. parameters and hypotheses is expressed in the terms of these distributions. The inferential process works by weighing one distribution (the ‘prior’) with another (the ‘likelihood’) and ending up with a third (the ‘posterior’). In the following presentation, we avoid the mathematics of how this process works, and instead focus on building a visual intuition^3^ of it; Etz and Vandekerckhove (2017) provide an accessible introduction to the computations for the interested.

#### The prior

The first component, the prior distribution, should incorporate all previous information – before seeing the data – about where the parameters might lie. Priors nudge the inference toward values that are credible. If this seems like an odd thing to do, bear in mind how we intuitively weigh evidence based on how extraordinary a claim it is supposed to corroborate. For example, we are much more prone to believe that smokers have a higher incidence of lung cancer than non-smokers, compared with smokers having better extrasensory perception abilities than non-smokers. This information would be included in the prior, so that our analysis would need less evidence to support the former than the latter.

Besides being required to obtain the posterior distribution, priors give researchers several advantages. The first of these is actually being forced to consider what is already known and expected of the phenomenon under study. Another prominent benefit is reducing *overfitting*; learning too much from the idiosyncratic properties of the data. When this happens, one is fooled into thinking that the model describes the regular, recurrent features of the phenomenon, when in fact it only describes the sample at hand (McElreath, 2017; Yarkoni & Westfall, 2016). Priors can thus ‘regularise’ our inferences: When we observe overly optimistic or pessimistic estimates (e.g. problematic measurements), they are weighted by the prior, hence distorting the analysis less and improving out-of-sample prediction. Other benefits of including prior information include helping circumvent the problem of non-identification in complex models (e.g. McElreath, 2016, p. 150).

It may seem like a daunting task to quantitatively describe prior information, and sometimes it truly is. In the end, the investigator must be equipped to defend the prior to a sceptical audience, who may have very different views of what can be considered reasonable (although one should aim to appease them with appropriate sensitivity analyses; Depaoli & van de Schoot, 2017). Still, when researchers interpret traditional analyses as posterior probabilities, it is often left implicit that they are assuming absolutely nothing of the phenomenon under investigation is known in advance. This is of course practically always false, and when little is known, one could carefully choose a prior which reflects that (see discussion on informativeness below).

**Figure 1.**
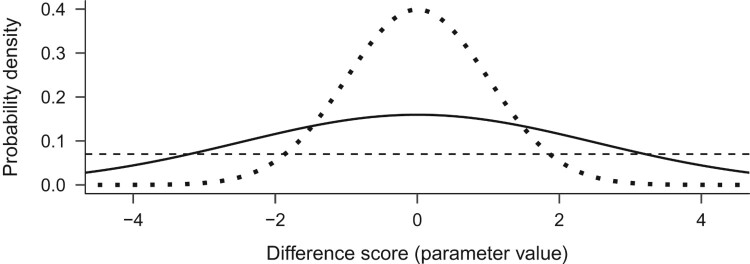
Three alternative priors, with varying informativeness. Dotted line depicts N(0, 1), solid N(0, 2.5), and dashed a uniform distribution.

Setting the prior can start from a very simple task, agreeing that impossible values are impossible: Our questionnaire had a scale of 1–5, so values of change larger than four and smaller than minus four are not possible. Further, we usually know how our measures behave in similar situations. It is easy to conjecture that small changes are more probable than very large ones in most if not all intervention contexts, and good reasons exist to assume the change scores approximate a normal distribution (for a maximum entropy justification, see McElreath, 2016, pp. 272–275). For simplicity, let us presume that the standard deviation will be one, making the measure coincide with Cohen’s d^4^. We could say that most changes are between ±1 (recall from earlier that the maximum change is four) and that few are more extreme than ±3. This information can be represented by a normal distribution with mean zero, and a standard deviation of 1, which is denoted N(0, 1). Thus, by the ‘empirical rule’ of normal distributions, 68% of effects would range between ±1, 95% between ±2 and 99.7% between ±3. We can use this distribution, visualized with a dotted line in Figure 1 as our prior.

Note that priors can vary as to their informativeness, and if they assert more specific effects, they affect the results more. The above is an example of an *informative* prior, albeit a quite weakly informative one. If we wanted a less informative prior, we could increase the standard deviation of the normal, or replace it with a Cauchy^5^ distribution, making the distribution flatter and thus more permissive of extreme events. Researchers should use existing evidence of similar interventions in similar populations to form informative priors, if they choose to use informative rather than non-informative ones. Alternatively, if we did not want to use prior information, we could set a *non-informative* prior, which states that all changes are as plausible a priori (represented by the horizontal line in Figure 1). This often results in the same numerical value as in frequentist estimation, but with a very different interpretation.

#### The likelihood

Next, in the data analysis phase, we multiply our chosen distribution with the likelihood. The likelihood represents the observed evidence itself; what the data tells us. It is the probability of data conditioned on different parameter values, multiplied by a constant.^6^

**Figure 2.**
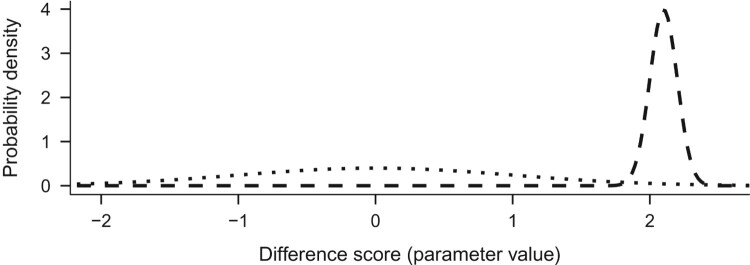
Prior (dotted) and likelihood (dashed) distributions.

Suppose we observed an increase of autonomous motivation score by a whopping 2.1 on average in a group of 100 people. The likelihood of this data, as a result of our chosen likelihood model, is represented by a normal distribution with a mean of 2.1 and a standard deviation of $\frac{\text{SD}}{\sqrt{n}}$ (see Dienes (2008), p. 93). Figure 2 presents the prior we defined earlier, N(0, 1), with the likelihood.

#### The posterior

**Figure 3.**
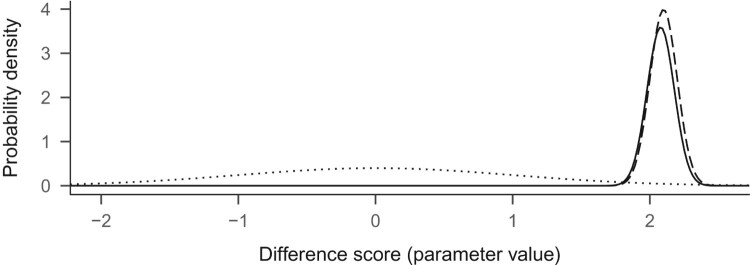
Prior (dotted), likelihood (dashed) and posterior (solid).

When the likelihood is multiplied with the prior, we end up with an updated view of the world, known as the posterior distribution. Think, for a moment, about the resulting values: multiplying something by zero gives zero, so the prior-times-likelihood combination is zero for all values except for the area from about 1.9 to about 2.4. The resulting posterior distribution is presented as the solid line in Figure 3.

As we can see, the prior nudged the posterior slightly to the left of the likelihood. Had the prior been flat, the posterior would have looked identical to the likelihood. Also, the more observations we have, the more prominent the likelihood is, and the less the prior matters. The posterior distribution as a whole is our estimate, but we could compress this information and report just the value with highest probability density like is often done with frequentist point estimates. On the other hand, the uncertainty around the estimate is usually crucial; we could present this by reporting the ‘credible interval’. A common choice for the credible interval is the central X% of the posterior distribution. For example, for the 95% credible interval, one could take the range between the 2.5 and 97.5 percentiles.

Note how frequentist confidence intervals often get intuitively confused with credible intervals. A 95% confidence interval for a mean tells you that 95% of intervals obtained from the sampling process would contain the population mean. However, any particular observed confidence interval either does or does not include the population mean; i.e. the probability of a given confidence interval containing the mean is either 1 or 0, not 95% (Morey, Hoekstra, Rouder, Lee, & Wagenmakers, 2015).

To obtain the posterior distribution, Bayesians usually use a method known as Markov Chain Monte Carlo (MCMC) (Ravenzwaaij, Cassey, & Brown, 2016). They do this because mathematically exact solutions are difficult or impossible to find in many applied cases. The MCMC method simulates the posterior by drawing random samples from the distribution. We will not go into details here, but suffice it to say that the more samples are drawn, the more accurate the result.

### Bayes factors

A Bayes factor $BF_{10}$ is the weighted ratio of two likelihoods. For simple point hypotheses, it is the likelihood of data given H1 divided by the likelihood of data given H0, commonly used in Bayesian hypothesis testing. It answers questions such as ‘Given the data, how many times more likely is a change of 0.5 compared to a change of zero’.

For simple models with so-called conjugate priors, which we will not delve into here, BFs can be very useful, but many applications have technical aspects which raise concerns. Some of these relate to using default priors, others to placing all prior mass to a single point; see e.g. Gelman and Rubin (1995), Robert (2016), and pages 182 and 193 in Gelman et al. (2013). We will not focus on BFs in this tutorial. For an accessible introduction to Bayes factors in health psychology context, we would like to direct the reader to Beard, Dienes, Muirhead, and West (2016). Dienes (2008) is a compact general introduction to the topic. In addition, Rouder, Morey, Verhagen, Province, and Wagenmakers (2016) shows some motivating examples behind the reasoning, Schönbrodt and Wagenmakers (2017) presents a design analysis perspective using BFs, and Etz (2015) is a practical guide to BFs in linear regression using R. Recently, the R package Bridge sampling (Gronau & Singmann, 2017) has been developed to deal with technical challenges in calculating BFs.

## The R environment for statistical computing

This tutorial will introduce Bayesian data analysis using the R environment for statistical computing (R Core Team, 2017). We focus on the R language for several reasons. First, with increasing demands for transparency and reproducibility in science, it is becoming increasingly important to plan work so that other researchers (and the future you) can understand what precisely was done to obtain the results (Munafò et al., 2017; Vuorre & Curley, 2017). Such reproducibility and transparency of communication is best achieved by doing statistical analyses using a programming language, instead of a point-and-click interface because by necessity each step in the former option is saved into the programming script that runs the analyses. This is reminiscent of the common practice of saving SPSS syntax for analysis, which however often omits e.g. changes in variable types in the graphical interface. Second, Bayesian data analysis is an extremely flexible tool, and for this reason has not yet been implemented to a satisfactory degree in point-and-click software (but see the JASP and jamovi programs: JASP Team (2017) and jamovi project (2017)). Finally, R is not only widespread and completely free of charge but in addition produces analysis scripts which can be opened by any text editing software, which contributes to the ideal of openness in science.

We have provided an introductory R tutorial elsewhere,^7^ but below reiterate the key points to allow the reader to follow this tutorial independently. For a deeper understanding of the R language, many online materials discuss the use of R in both written (Navarro, 2015; Phillips, 2017; Vuorre, 2016) and video (Phillips, 2015) formats.

### Installing R and RStudio

**Figure 4.**
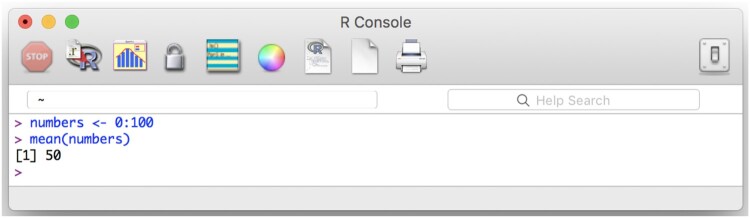
The R console. This figure shows how to assign (R uses the left arrow, <-, for assignment) all whole numbers from 0 to 100 to a variable called numbers. Computer code can often be read from right to left, so the first line here could be read as ‘integers 0 through 100, assign to numbers’. We then calculated the mean of those numbers by using R’s built in function, mean().

The R programming language can be downloaded for free for Windows, Mac, and Linux operating systems,^8^ and installed like any other application. To use the R programming language, one needs to access it through a console, which is a text-based input-output interface – the user types in and executes input, the program returns output. The R console application can be opened like any other application on your computer, after it has been installed. We show the R console in Figure 4 along with a few simple commands for saving numbers into a variable and computing their mean. You can type out the commands from Figure 4 on your own computer and execute them by pressing Return (Mac) or Enter (Windows).

However, the use of R is made significantly easier (and more pleasant, we suggest) by the popular RStudio (RStudio Team, 2016) Integrated Development Environment (IDE), which we strongly recommend. RStudio provides many helpful features for conducting statistical analyses (and more) with the R language, and can be downloaded free for Windows, Mac, and Linux.^9^

### Data analyses are saved as scripts

**Figure 5.**
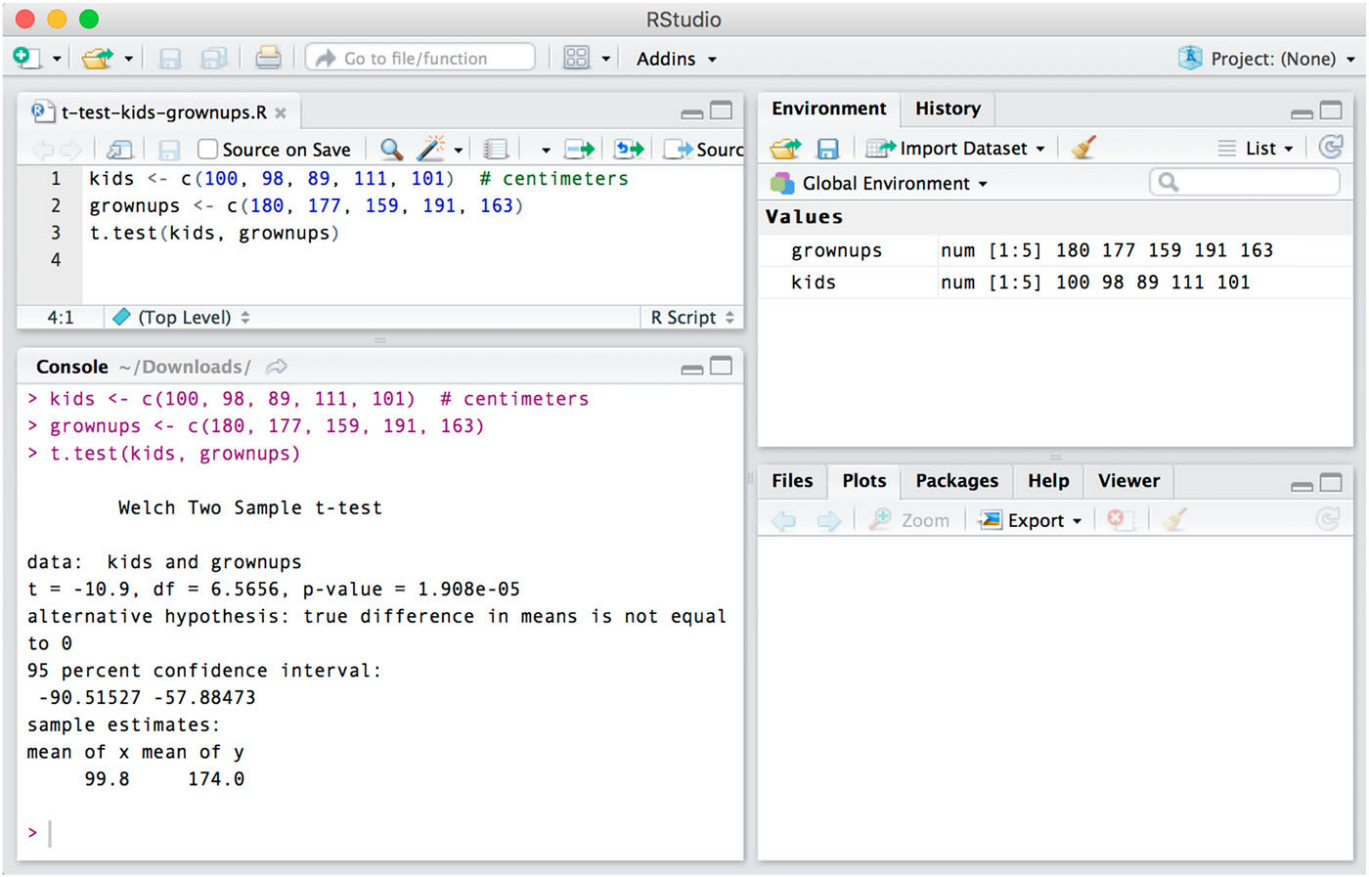
RStudio with its text editor and R console (upper and lower left panels, respectively). The three lines of code saved into the R script ‘t-test-kids-grownups. R’ shows how to save numbers into variables, and then conduct a t-test between the variables.

Although R’s data analysis functions, such as loading and transforming data, creating figures and estimating statistical models, can be written and executed directly in the console, it is important that you save these commands into scripts. R scripts are files that contain the functions of a statistical analysis in the order in which they should be executed. An example R script is shown in Figure 5, where the R script for doing a *t*-test between two groups is shown in RStudio’s text editor panel in the upper left corner. When these lines of the script are executed (move the text cursor onto the appropriate line and press Command + Return (Mac) or Control + Enter (Windows)), their output is printed in RStudio’s R console panel (bottom left). Whatever variables and figures are created in the script will be visible in the upper right and lower right RStudio panels, respectively. To create an R script, click File → New File → R Script in RStudio. We suggest you follow this tutorial by typing the commands into a new R script.

### Basic R commands

Figure 4 showed two basic R functions (saving numbers into a variable, computing the mean of the numbers inside a variable). Figure 5 shows a function to conduct an independent samples *t*-test. All R operations are based on functions, which can be identified by the fact that they are followed by parentheses (e.g. mean() for computing a mean) and arguments that are entered inside the parentheses (e.g. numbers). In this tutorial, instead of showing screenshots for each line of R code, we show the code inline, which for Figure 4 would look like this:

numbers <- 0:100 mean(numbers) ## [1] 50

In the above code listing, the output of the last function is prepended with two **#**s to separate it from the input functions, which are not prepended. The R programming language contains a great number of useful functions, but the true power of R is realized in user-contributed packages, which contain many more functions to extend R’s functionality. To obtain these packages, and their associated functions, users must first install the packages. In this tutorial, we will illustrate Bayesian data analysis with R functions contained in the brms (Bayesian Regression Models using Stan) package (Bürkner, 2017; Stan Development Team, 2016a). To install R packages, you simply call the install.packages() function in the R console, with the name of the desired package (in quotes) as the argument^10^. To start with the tutorial, install the brms package^11^ by running the following command:

install.packages("brms")

You should only install packages once. That is, the next time you run this code, you should not re-install the package, as it will be saved on your computer. Next, you will need to read the appropriate data file into R’s workspace. There are many functions in R that read data from files, and we recommend using functions found in the tidyverse package (Wickham, 2016)^12^.

To read a data file into an R object that you can use in the current R session, you need to use a function to read a file on your computer’s hard drive. With this tutorial, we have provided a data file called motivation.csv. You should place it somewhere where you can easily find it. Here, we assume that you are writing an R script, and you should place the data file in the same directory as the R script. Then, assuming that your R working directory^13^ is the directory with both these files, you can call the read_csv() function, and pass the data file’s name as an argument. The first line in the following code listing loads the tidyverse package’s functions so the read_csv() function is available.

library(tidyverse) d <- read_csv("motivation.csv")

d is now an object in the R workspace that you can use for visualization, modeling, and more.

## Bayesian inference in practice

Having introduced the basic concepts of Bayesian inference, we can now apply them in practice. In summary, a practical Bayesian inference can be thought to consist of five steps of analysis (Kruschke, 2014), described in Table 1. We now turn to Step one of Table 1 and describe the data used in this example.

**Table 1.** Five conceptual steps of Bayesian data analysis.

Step	Procedure
1	Identify data relevant to the research question.
2	Define a descriptive model, whose parameters capture the research question.
3	Specify prior probability distributions on parameters in the model.
4	Update the prior to a posterior distribution using Bayesian inference.
5	Check your model against data, and identify possible problems.

Note: Adapted from Kruschke (2014, p. 25).

### Step 1: identifying relevant data

The first step of Bayesian data analysis, as it is in any analysis, is to identify the data, because we wish to infer something about the world based on data. The example data are illustrated in Table 2, and described in more detail above. This table shows the variables available to use in the statistical model.

**Table 2.** Data set from example intervention study.

ID	intervention	item	time	value
1	1	intrinsic_a	0	5
1	1	intrinsic_b	0	4
1	1	intrinsic_c	0	4
1	1	intrinsic_d	0	4
1	1	identified_a	0	5
1	1	identified_b	0	2

Note: The data are in the standard long format, where each observation (questionnaire response) is in its own row. This format is expected by the regression equation (below) and is in contrast to wide-format data where an individual’s repeated measures are on a single row. Value is the actual numerical response, and the ID and item variables specify whose response it is and to which specific questionnaire item. Missing values are indicated by NA.

The primary research question relates to the extent to which the intervention causes changes in autonomous motivation. We, therefore, identify the output variable in the data as the individuals’ survey responses which relate to autonomous motivation. The main input variables are intervention (coded as 0 and 1 for the control group and intervention group, respectively) and time (coded as 0 and 1 for baseline and post-intervention, respectively). Having operationalized the concepts as variables in the data, we can next define the statistical model.

### Step 2: define the statistical model

Our statistical model will consist of defining a likelihood function for the outcomes, which are the survey responses. For each row i and person j in the dataset, the unique survey response is denoted as $Y_{ij}$. As is usual for most regression models, we define that the outcomes follow a Gaussian (i.e. ‘Normal’) distribution with two parameters, μ for mean, and $\sigma^{2}$ for residual variance. The outcome distribution or the model of the outcomes is^14^

$$Y_{ij}\overset{iid}{∼}N(\mu_{ij},\sigma^{2}),$$

where the $\overset{iid}{∼}$ symbol denotes ‘independently and identically distributed’ (in what follows we drop the *iid* to simplify notation, but continue to assume it). The next step is defining the linear model for the parameter(s) of the Gaussian distribution. The most basic model would be to model the mean as a linear function of time and intervention. However, this model would ignore the fact that the *Y*_*ij*_ are not independent, because each person provided two observations: The data consist of repeated measures of individuals over time.

The second reason for not using the simple model is the fact that each participant answered eight survey items. For the example model in this tutorial, we solve the second complication by averaging the outcome for each person, at each time point, over the eight different questionnaire items – as is commonly done. However, averaging is in no way necessary and the model can be easily extended to handle multiple response scales, but for this introductory tutorial, we do not discuss that extended model.

There are many ways to aggregate data in R, and here we use a common strategy where summarizing functions are applied to ‘groups’ in the data (Wickham & Francois, 2016). In the following code listing, we create a new variable called avg by taking the data frame d, then grouping it by ID, intervention, and time (second line). The effect of this code is that any following summarizing operations are applied to combinations of these grouping factors. The %>% symbol is used to pass results from one line to the following one, which eschews the need to save intermediate results. The third line calculates the mean of value for each of the groups defined in line two. na.rm = TRUE means that the mean should be calculated after removing missing values (if left in, any group with any missing values would have a missing value as the mean.) The fourth line removes the grouping information from the data frame.

avg <- d %>%   group_by(ID, intervention, time) %>%   summarize(value = mean(value, na.rm = TRUE)) %>%   ungroup()

The data in this aggregated form is illustrated in Table 3, and we now understand ${\text{Y}}_{ij}$ to mean the average motivation scale response over the 8 items for person j on row i.

**Table 3.** Data set from example intervention study.

ID	intervention	time	value
1	1	0	4
1	1	1	4
2	1	0	4
2	1	1	4.38
3	1	0	4
3	1	1	4

Note: Data aggregated over the questionnaire items, resulting in two observations per person.

Traditionally, to address the fact that the responses are correlated within people across the two time points, researchers have commonly turned to the repeated-measures ANOVA model. However, we take a more general approach, based on multilevel modeling (Bolger & Laurenceau, 2013; Gelman & Hill, 2007). Multilevel modeling – sometimes called hierarchical or linear mixed effects modeling – is an increasingly popular method for modeling data which consist of non-independent observations, such as repeated measures in treatment evaluation studies. The key assumption of multilevel modeling is that the lower-level observations (individual survey responses) are clustered within upper level units (participants).

Multilevel models have many benefits over the traditional rm-ANOVA approach, such as allowing unbalanced data^15^, continuous predictors, and categorical outcomes (Bolger & Laurenceau, 2013; Gelman & Hill, 2007; Jaeger, 2008; McElreath, 2016). Importantly, these models do not require data to be collapsed to person- or cell-means and thereby allow estimating the extent to which the effects (co)vary in the population of individuals. We, therefore, specify a regression model which accounts for the repeated measures by including an intercept term for every individual (i.e. a ‘varying intercepts model’; Gelman and Hill (2007)):

$$\mu_{ij}=\alpha_{ij}+\beta_{T}\text{tim}{\text{e}}_{ij}+\beta_{I}\text{interventio}{\text{n}}_{ij}+\beta_{IT}(\text{tim}{\text{e}}_{ij}\times \text{interventio}{\text{n}}_{ij})$$

This equation shows that we model autonomous motivation on an intercept (*α*, more on which later), and regression coefficients for time ($\beta_{T}$), intervention group ($\beta_{I}$), and their interaction ($\beta_{IT}$). These latter three parameters capture our research questions about the effects of time and intervention on the response variable, and the difference of the effect of time between the intervention groups (the interaction term), respectively. With respect to the research question, we are most interested in $\beta_{IT}$, which quantifies the extent to which the effect of time differs between the two groups. The effect of time for the control group is defined by $\beta_{T}$ (because the control group is used as the ‘reference’ group by coding it as zero). Similarly, $\beta_{I}$ quantifies the effect of intervention at time 0.

The subscripted $\alpha_{ij}$ parameter demands more attention: It reflects J (number of persons in the study) intercepts, and therefore assigns an intercept to each person j – which are therefore called ‘varying intercepts’. The person-specific intercepts are modeled as draws from a distribution:

$$\alpha_{j}∼N(\beta_{0},\tau_{0})$$

This latter equation reveals the ‘multilevel’ nature of the model: Each person j’s intercept is assumed to be normally distributed on a mean intercept $\beta_{0}$, and the spread of these intercepts is captured by the standard deviation $\tau_{0}$. In other words, we can consider that there are two levels of intercepts; the person-specific intercepts are draws from an upper level distribution, whose mean describes the average intercept. In frequentist literature on multilevel modeling, the average effects ($\beta_{0}$) are often known as ‘fixed’ effects, and the lower- or person-level intercepts are known as ‘random’ effects because they are assumed to vary randomly as defined by the normal distribution. However, in the Bayesian framework, it is less meaningful to call only one of these parameters ‘random’ (Gelman & Hill, 2007, p. 245). Correspondingly, we describe the ‘random’ parameters as varying – for example, varying between participants – and the ‘fixed’ parameters with their corresponding level of analysis. Here, the ‘fixed’ intercept ($\beta_{0}$) refers to the average person’s intercept, or similarly to the expected intercept in the population, as in frequentist ML modeling. We, therefore, refer to the ‘fixed’ effects as ‘population-level’ effects.

### Step 3: specify prior information

In the Bayesian framework, all parameters which are not themselves modeled are assigned prior probability distributions^16^. These ‘priors’ describe the available information about the parameters before seeing new data. The current model has six unmodeled parameters: The four population-level regression coefficients (including the intercept $\beta_{0}$), the standard deviation parameter of the varying intercepts ($\tau_{0}$), and the standard deviation of the data distribution σ (which, when squared, is sometimes called the variance of the residuals).

How should researchers specify prior information about the to-be-estimated quantities of their statistical models? Above, we distinguished between informative and non-informative priors and discussed how inference may benefit from using priors that gently guide the inference toward credible values (Gelman et al., 2013; McElreath, 2016). When defining a prior for estimating intervention effects on the autonomous motivation for PA among youth, a health psychologist might turn to existing research evidence. This is a clear advantage over the frequentist approach, where the researcher appears to not have much clue about the size of the effect based on previous studies that could be considered in data analysis. In our case, the evidence may inform us that on the whole, school-based PA interventions among older adolescents result on average in modest effects at best (Hynynen et al., 2016), and that experimental evidence on self-determination theory-based interventions has been scarce (Ng et al., 2012; Ryan & Deci, 2017).

Additionally, we would need to rather take into account the evidence of interventions of similar content, dose, and intensity, with about a similar six weeks of follow-up, which would correspond closer to our study design, compared to other types of interventions. Such studies are rare. Hence, we would be advised not to set a highly informative prior. We, therefore, begin our analysis using minimally informative priors (Kruschke, 2014).

**Figure 6.**
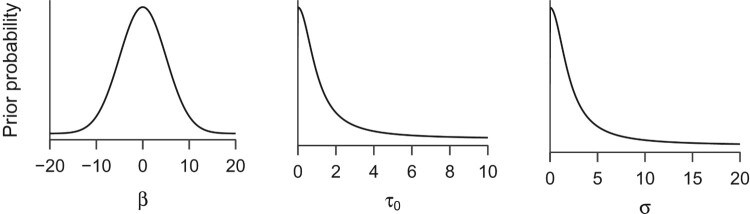
Prior probability distributions for Model 1 in the tutorial. The left panel shows the prior distribution which is assigned to all regression coefficients β. Middle panel shows the prior distribution of the standard deviation parameter of the person-specific intercepts. Right panel shows the prior distribution for the residual standard deviation.

These priors assign credibility to a wide range of parameter values, but have their peak at zero, reflecting our mild assumption that greater (negative or positive) effects should be less plausible than ones near zero. For the four regression coefficients, we assign Gaussian distributions with mean 0 and standard deviation 5, shown in the left panel of Figure 6. Although the effects cannot be greater than four – because the ratings are made on a 1–5 scale – defining a prior with strict boundaries in addition to the smooth decline of the Gaussian density is outside the scope of this tutorial (Gelman et al., 2013).

β∼N(0,5).

The prior distribution for the standard deviation of the varying intercepts ($\tau_{0}$; middle panel of Figure 6) assigns maximum a priori probability for zero, and decreasing plausibility toward greater values. This distribution is a positive only Cauchy distribution with scale 1 (Gelman, 2006). In this case, the prior explicitly reflects our mild a priori assumption that smaller values of between-person heterogeneity are more likely than larger ones.

$$\tau_{0}∼Cauchy^{+}(0,1).$$

Finally, the right panel of Figure 6 shows a positive only Cauchy with scale 2, which is used as the prior distribution for the standard deviation of the residuals (σ). This distribution is so broad that it has next to no influence on the estimated parameter values.

$$\sigma ∼\text{Cauch}{\text{y}}^{+}(0,2).$$

### Step 4: Bayesian inference

After the first three conceptual steps of Bayesian data analysis in Table 1 (Kruschke, 2014), we can now use Bayesian inference to update the prior distributions to a joint posterior distribution that describes the plausible parameter values after seeing the data. We have above described the theory of Bayesian updating, and also noted that for complex problems with many parameters, analytical (i.e. mathematically exact) solutions might not be available. We, therefore, turn to computer methods for estimating the model. These computer methods are available in the R package brms, which we installed above (Bürkner, 2017). To make the functions of brms available in the current R session, we need to ‘load’ the package in the beginning of the data analysis script^17^:

library(brms)

We must then translate the mathematical model described above into a form that R can understand. To do this, we specify the model in R’s modeling syntax (which is extended by brms to Bayesian regression models).

#### R modeling syntax

R’s modeling syntax is a powerful language for expressing mathematical models in a form that can be passed to various functions for estimation. Generally, for response variable(s) *Y*, and input variable(s) *X*, models are written as

Y ∼ X1 + X2 + X1:X2

which can be read as ‘Y is modeled on X1, X2, and their interaction’. The syntax also allows a shortcut for including the main effects of two variables and their interaction

Y ∼ X1 * X2

which implicitly expands out to include all three predictor terms. The model syntax also implicitly adds the intercept term, which can be explicitly included with a 1:

Y ∼ 1 + X1 * X2

Finally, we must add the varying coefficients. These are added by a two-sided formula, whose predictor terms (intercept in the current example) are on the left-hand side of a |, and whose grouping terms (participants, identified by variable ID) are on the right hand side.

Y ∼ X1 * X2 + (1 | ID)

In the previous code listing, the equation in the parentheses means that intercepts (1) vary between the clusters (participants, as identified with the ID variable in the data). For the current example model, we specify the model using the appropriate variable names, and wrap the model formula into brms’ bf() function.

model_1 <- bf(value ∼ 1 + time * intervention + (1 | ID))

model_1 is now an R object that can be passed on to the estimation function. But first, we specify the prior distributions.

#### Specifying priors

Next, we introduce how to set priors to the regression model, but readers who wish to estimate the model with brms’ default priors^18^ can initially skip this section. Given the saved model object, we can use brms’ helper function get_prior() to show which parameters can be assigned prior densities.

get_prior(model_1, data = avg)

This function returns a table showing which parameters (or groups of parameters) can be assigned priors (relevant output is shown in Table 4). To assign the prior distributions discussed in the previous section, we use brms’ function prior() whose first argument must be an unquoted character string describing a distribution in Stan language (Stan Development Team, 2016b). For example, the N(0,5) distributions for the regression coefficients are defined with

**Table 4.** Possible (classes of) parameters that can be assigned priors in the example model.

Class	coef	group
b	intervention	
b	time	
b	time:intervention	
Intercept		
sigma		

Note: Only relevant output shown.

prior_betas <- prior(normal(0, 5), class = "b")

where the class = “b” indicates that this distribution should be assigned as a prior to all the ‘betas’, or regression coefficients^19^. The two Cauchy priors for the standard deviation parameters are created with

prior_tau <- prior(cauchy(0, 1), class = "sd") prior_sigma <- prior(cauchy(0, 2), class = "sigma")

and we can then combine all these priors into one variable with R’s c() function

prior_1 <- c(prior_betas, prior_tau, prior_sigma)

The object prior_1 now contains all six prior distributions, and can be passed on to the estimation function.

#### Fitting the Bayesian model

We have now defined the model’s regression formula, which is saved in model_1, and it’s associated prior distributions, saved in prior_1. We are therefore ready to estimate the model. To estimate the model – more technically, to draw samples from the model’s posterior distribution – we use the brm() function:

fit_1 <- brm(model_1, avg, prior = prior_1)

Brms’ brm() is a powerful function whose input arguments are a model formula (model_1), a data frame (avg), an optional prior definition (prior_1), and various optional arguments (see ?brm). The function then translates the arguments into a Stan model and instructs the Rstan package to draw samples from the posterior distribution (Stan Development Team, 2016a). By default, brm() runs 2000 iterations over four MCMC chains, and uses the first half of each chain to adjust the underlying algorithm, resulting in 4000 random draws from the posterior distribution of the model. When this function is executed, brms will first report that it is compiling a C++ model, which may take up to a minute for complex models, and then reports on the progress of drawing samples, and finally produces an object (here saved to fit_1) with all the information about the estimated model. This object can then be used in other functions to output numerical and graphical summaries of the estimated model.

#### Interpreting the model’s output

To print the estimated parameters of the model in R’s console, you can use the summary() function:

summary(fit_1)

We first interpret the population-level effects of the output (Table 5). This table reports the posterior mean and standard deviation (the analogous frequentist quantities are the parameter’s point estimate and standard error, respectively) for each of the four population-level regression coefficients. First, the intercept’s row describes the plausible values of the motivation response at time 0 and intervention 0 (first time point, control group) for the average person. Estimate is the mean of the posterior distribution, and corresponds to the frequentist point estimate: We expect the average person to report a baseline motivation of 3.69. However, the 95% credible interval (indicated by its lower and upper bounds) shows that this value could be as low as 3.29 or as high as 4.10. Est.Error is the standard deviation of the posterior distribution.

**Table 5.** Population-level effects of the estimated model.

Parameter	Estimate	Est.Error	*l*–95% CI	*u*–95% CI	Eff.Sample	Rhat
Intercept	4	0	3	4	885	1
Time	0.09	0.15	−0.20	0.38	2247.70	1.00
intervention	−0.09	0.26	−0.63	0.44	803.84	1.01
time:intervention	0.10	0.18	−0.27	0.45	2247.06	1.00

Note: Estimate is the posterior mean and Est.Error the posterior standard deviation.

**Table 6.** First six rows of random samples from the posterior distribution of the model’s population-level effects.

b_Intercept	b_time	b_intervention	b_time:intervention	delta
3.80	0.11	−0.44	0.05	0.16
3.47	0.35	−0.09	−0.14	0.21
3.39	0.34	−0.05	−0.02	0.32
3.48	0.04	0.15	0.08	0.11
3.29	0.27	0.31	0.01	0.28
3.40	0.13	0.20	−0.14	0.00

Note: The samples are obtained from the MCMC sampling procedure. Delta is the posterior distribution of the effect of time in the intervention group, which is the sum of b_time and b_time:intervention.

Eff.Sample describes the number of efficient samples from the posterior distribution; these are the number of (roughly) independent samples obtained from the distribution, while accounting for their autocorrelation. Rhat is the Rubin–Gelman convergence diagnostic, and should be 1.00 for accurate estimates of the posterior distribution (Gelman et al., 2013, pp. 285–288).

Next, time describes the plausible values of change in motivation for the control group. Ninety-five percent of the most plausible values of change are between −0.20 and 0.38: The point estimate of 0.09 is quite small in light of this uncertainty, and we are therefore unable to conclude with confidence that the control group changed much between the two time points. The intervention parameter describes the plausible magnitudes of the intervention’s effect at time 0.

The most important parameter with respect to the research question is the interaction term time:intervention. This parameter’s point estimate (posterior mean) is small, and the relatively wide 95% Credible interval, ranging from −0.27 to 0.45 suggests that our knowledge about the parameter’s location is uncertain. In other words, given the prior information and the data, we have learned relatively little about the effectiveness of the intervention, and our uncertainty about the parameter is considerable: We are unable to assert with confidence that there is a meaningful difference in how the two groups changed over time.

**Figure 7.**
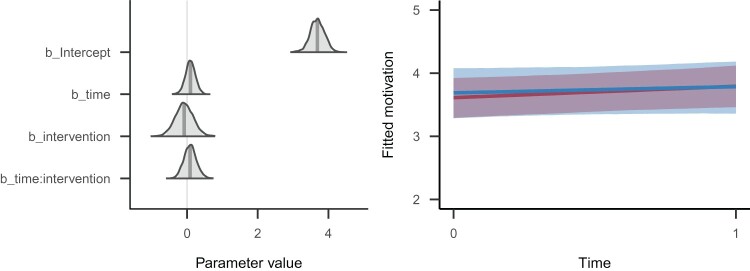
Left panel: Density curves of the posterior distributions of the four population-level regression parameters. The shaded area indicates the 95% Credible Interval, and the vertical line indicates the posterior mean. The density curves are estimated from MCMC samples, and slightly smoothed for the figure. Right panel: Trajectories of change across time for the two intervention groups (blue: control group, red: intervention group). Each line denotes the posterior mean regression line for that group, and the surrounding shades are the 95% Credible Intervals for the regression lines. The code for creating these two figures can be found in the complete code listing for this tutorial.

We have also illustrated the model’s estimated parameters and fitted response values graphically in Figure 7. The left panel of this figure illustrates the estimated parameters from Table 5 graphically as (slightly smoothed) probability densities. This figure was created using the bayesplot package’s (code not shown) mcmc_areas() function (Gabry, 2017). The right panel displays the implications of the model’s posterior distribution in the scale of the data, created with brms’ marginal_effects() function (code not shown).

Given these numerical estimates (representing the model’s posterior distribution), we are now in the position to answer the research questions. We asked: ‘To what extent does the intervention affect autonomous motivation?’ As first pass, we have interpreted the population-level effects in Table 5, whose time:intervention parameter described the current state of knowledge about that parameter: The point estimate was positive, yet very small in the context of the considerable uncertainty, represented by the bounds of the 95% credible interval. In sum, this estimated parameter suggested to us that there was not much difference in how the two groups changed across time. However, note that there is no parameter describing the magnitude of change in the intervention group.

Fortunately, the matrix of posterior samples represents a joint posterior probability distribution, and we can use it to create posterior distributions for quantities that answer further questions. More specifically, we need to obtain the posterior distribution of $\delta =\beta_{T}+\beta_{IT}$, which quantifies the rate of autonomous motivation’s change over time for the intervention group. This can be simply calculated from the posterior samples (see Table 6).

**Figure 8.**
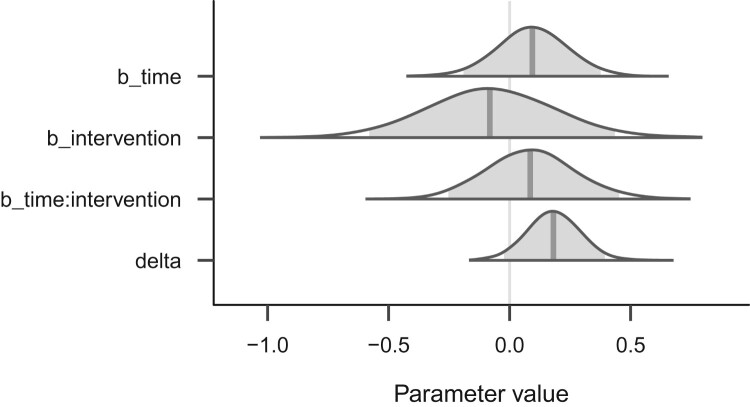
Posterior distributions of the three main population-level regression coefficients, and the transformed parameter δ (delta), which denotes the effect of time in the intervention group only.

This quantity of interest δ can now be summarized and visualized for drawing inference about the magnitude of time’s effect in the intervention group. Although we could not conclude with confidence that the control and intervention groups changed differently over time, we may still be interested in the intervention group’s magnitude of change. To address this question, we repeat the left panel of Figure 7 in Figure 8: The bottom row of this figure (‘delta’) shows the posterior distribution of the intervention group’s change over time, which appears modest (the point estimate, posterior mean, is 0.18). Additionally, this modest value is qualified by relatively great uncertainty, which is represented by the spread of the posterior distribution (the 95% credible interval is [−0.04, 0.41]).

We can also calculate the proportion of the posterior density that is above zero to approximate the posterior probability that the effect is positive.^20^ The answer turns out to be that 95.40% of the density lies above zero, and we can therefore assert 95.40% confidence that the effect is positive. This posterior probability is numerically analogous to the frequentist one sided *p*-value (Marsman & Wagenmakers, 2016), but notice that we can directly interpret the posterior probability as asserting confidence, or subjective probability, in the sign of the parameter. We should not, however, interpret this value as quantifying the evidence for, or probability of, a quantitative hypothesis about the data – such questions are better answered by Bayes Factors, which are outside the scope of this tutorial.

#### Assessing the computational algorithm’s performance

In many applications of Bayesian statistics, such as one discussed here, the model’s posterior distribution is not analytically calculated, but rather approximated by an MCMC algorithm. Technical details of this algorithm are outside the scope of the current article (see Kruschke (2014); Ravenzwaaij et al. (2016)), but users should be familiar enough with it to assess whether the posterior approximation through MCMC sampling is adequate.

A ‘chain’ of MCMC draws is a random sequence of samples from the model’s posterior distribution. By default, the software used here returns four chains of 2000 samples each. Four chains are almost always adequate, but users may wish to increase the default number of samples for some applications. The first half of each chain is used to adjust the behavior of the sampler and is automatically discarded before the results are displayed. There are many methods for assessing the chains’ ‘convergence’ (the representativeness of the random sample), here we highlight two. First, as noted before, the Rhat quantity in the model’s summary output should be very close to 1. Values different from 1 suggest that more samples should be drawn from the posterior distribution.

**Figure 9.**
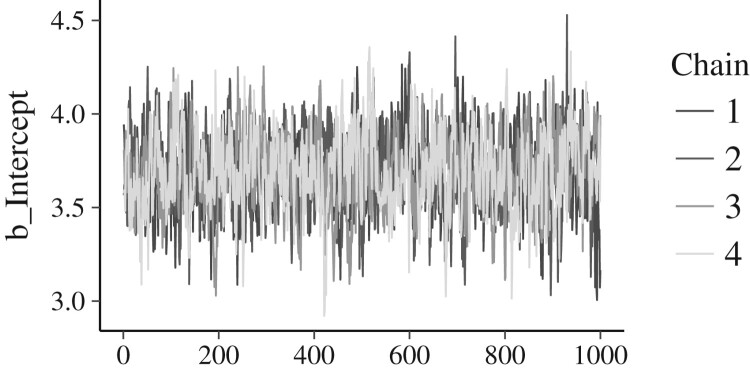
Traceplot of the model’s intercept. Each of the four chains is plotted in a different hue. The posterior samples (x-axis) are connected with a line; y-axis are the samples’ values. The four chains’ traces look highly similar, suggesting to us that the MCMC approximation has worked well. If the chains looked very dissimilar, we would be prompted to further investigate the model’s performance.

Another method of monitoring convergence focuses on visual inspection of the MCMC chains: The four chains of samples should look highly similar to one another, if they all are representative samples from the true posterior distribution. Figure 9 shows a ‘traceplot’: A visual representation of the four MCMC chains of samples from the model’s intercept’s posterior distribution. The four chains look highly similar, reassuring us of good performance. Dissimilar chains suggest that further investigation into the model’s performance is needed.

### Step 5: model checking

The goal of model checking is simple: After a model has been estimated, the modeler should ensure that the model captures the important features of the data, and that reasonable inference can be drawn. This process is analogous to that of all modeling endeavors: Colloquially, the model should ‘fit’ the data well. The topic of model checking is broad, and here we advocate and illustrate graphical model checking in the form of posterior predictive checks (Gelman et al., 2013, p. 143).

**Figure 10.**
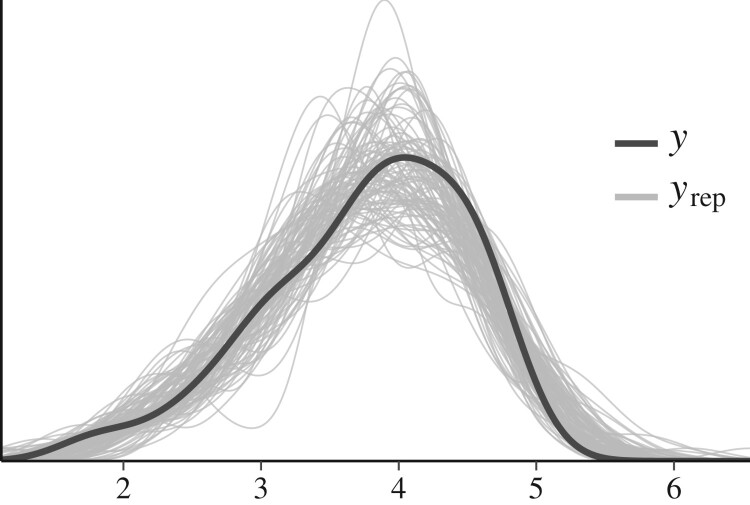
Graphical comparison of the actual data set to replicated data sets should reveal a very similar shape of the densities, if the model fits the data well. Here, we do not see serious problems with how the model seems to replicate the data (but note that we have not taken into account the natural 1–5 limits of the response scale, or that the raw responses are ordered categories).

Posterior predictive checks allow assessing whether the model’s predicted values are similar to the actual data. If the model fits the data well, the model’s predicted values and the data would look similar. brms provides helper functions for performing graphical checks (Bürkner, 2017; Gabry, 2017), which we use here. Although a complete review of this topic is beyond the scope of this paper, in Figure 10 we graphically compare the density of the data (*y*) to densities of 100 datasets that are simulated from the model ($y_{rep}$).

Although this figure doesn’t suggest serious problems with the model, we can see room for improvement. For one, we can see that because we have not included information about the natural limits of the data, the model’s replicated datasets suggest that values above 5 are possible. The model could be expanded to include this information. Additionally, we could have instead modeled the raw discrete ratings as an ordered variable, instead of modeling the averaged responses as a continuous variable, but this topic is outside the scope of this tutorial. However, these assumptions are common to many regression models which do not explicitly specify the data limits, or use aggregated responses instead of raw ordered categories, such as common ANOVA methods. Solutions and software are described in Saarela (2017), Saarela and Arjas (2015), and Bürkner and Vuorre (2018). We also show how to analyze the raw data with an ordinal logistic regression using brms in the Appendix.

In sum, based on the steps presented, the results of the estimation are as follows: given the model and the data, it is fairly unlikely that the intervention has an unintended negative impact on autonomous motivation. Furthermore, even quite large effects are plausible, but there is vast uncertainty regarding the effect, due to the small number of participants in this feasibility study.

### Summary of practical tutorial

In the above tutorial, we covered the five conceptual steps of Bayesian data analysis (Table 1; Kruschke (2014)). We hope to have shown that this extremely powerful and flexible *probabilistic* approach to statistical modeling is now available and relatively easy to start applying through the easy-to-use R interface to the Stan modeling language, brms (Bürkner, 2017; Stan Development Team, 2016a). The brms R package allows specifying models and priors for a wide range of models, from simple comparisons of two groups to more complicated multilevel analyses. Importantly, the flexible Bayesian approach brings with it the benefits of the Bayesian framework, highlighted above in our discussion about Bayesian inference.

## Ethics Statement

The study protocol was reviewed by an Ethics Committee of the Hospital District of Helsinki and Uusimaa (Decision number 249/13/03/03/2011). Participants were treated according to principles of the Helsinki Declaration, and were informed about their right to withdraw from the study at any point.

## Conclusions and recommendations

The aim of this tutorial article was to provide a brief overview of the Bayesian approach for beginners, accompanied by a hands-on demonstration of Bayesian methods and reasoning regarding intervention effects, using a small intervention study dataset with intervention and control arms. We have attempted to avoid overselling the approach and would like to emphasize that researchers should carefully consider what their objective – what they want to know or do – is, *prior* to choosing a suitable methodological approach. For example, a researcher may aim to control for long-run error rates of decisions. It may then be acceptable to use modern frequentist hypothesis testing (see Haig, 2016 for an approachable introduction) for differences in outcomes, between randomly assigned participants in treatment and control groups. Note that researchers still ought to be able to justify e.g. their alpha levels, instead of using same conventions for all situations (see Lakens et al., 2017).

One of the main advantages of the Bayesian approach to intervention evaluation is that it more fully makes use of all available information, including in the form of prior distributions. The prior distributions also function as an intuitive way to regularize inferences, in order to avoid overfitting. In general, Bayesian modelling encourages the researchers to explicate many assumptions behind the analysis, allowing for more thoughtful and thorough inferences. As pointed out in the introduction, major advantages become apparent in more complex models than the current, minimal pedagogical example.

Criticisms for adopting (exclusively) Bayesian inference have been voiced, too. Among the most prominent critics, a leading frequentist philosopher of statistics Deborah Mayo cautions against abandoning the error statistical approach to testing, which accommodates for a comprehensive model of cumulating knowledge from experiments (Mayo, 1996, 2013a, 2013b). One such criticism is that without an error statistical framework, it is difficult to evaluate how severely a claim has been tested. Readers interested in learning more of possible risks of a fully Bayesian philosophy of science may find Mayo (2018) useful.

Pitfalls and risks for aspiring Bayesians are presented in the ‘When to worry and how to Avoid the Misuse of Bayesian Statistics’ (WAMBS) checklist (Depaoli & van de Schoot, 2017), which we encourage embracing. In crude summary, researchers should understand how sensitive their models are to changes in assumptions, including priors. For this reason, transparent documenting and reporting of the research process, including sharing the analysis code for reproducible reports, is crucial for evaluating results. In the age of practically unlimited free space for supplementary files^21^ in e.g. the Open Science Framework website (http://osf.io), we strongly urge researchers to make use of such repositories.

Scientific thinking is crucial when health psychologists add Bayesian tools to their toolbox of statistical methods. There will be no universal, nor automatic, method to answer all inferential needs (Gigerenzer & Marewski, 2015). We urge researchers in the field to consider their research questions thoroughly (see e.g. Hand (1994) for advice) and investigate whether the conventional methods really provide them with the answers they are looking for.

## Figures and Tables

**Figure A1.**
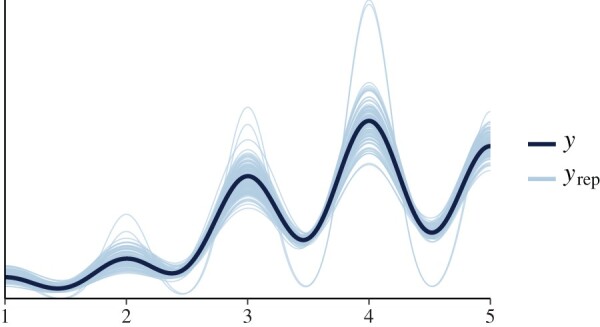
Posterior predictive check for the ordinal logistic model.

## Notes

1 Note how we do not find e.g. the probability of being wrong, or the hypothesis being false, or the probability of getting the same result in a replication study (Gigerenzer, 2004; Wasserstein & Lazar, 2016).

2 These parameters mean the same as in classical statistics. They work like control knobs for adjusting the heat of an oven or the volume of loudspeakers. For example, a normal distribution’s position on the x-axis is controlled by the parameter *mean*, and the spread by the parameter *standard deviation*.

3 See http://rpsychologist.com/d3/bayes/ for an interactive visualization of the interplay between the prior, likelihood and posterior.

4 The mean group difference divided by the standard deviation of the difference. See http://rpsychologist.com/d3/cohend/ for a visualization.

5 A Cauchy looks like the normal, but has thicker tails. Centered on zero with a scale parameter of 0.5, it would consider 50% of effects to be within 0.5 of zero, and the rest to be more extreme – possibly very extreme, as the probability of drastic effects such as d=10 never becomes so small that they could be considered practically impossible.

6 An accessible intro to the intricacies of likelihood is found in Etz (2017)

7 See http://blog.efpsa.org/2016/12/05/introduction-to-data-analysis-using-r/ for a comprehensive introduction to the basics of using R and R Studio.

8 https://cran.r-project.org/.

9 https://www.rstudio.com/.

10 The function will automatically install the desired R package to an appropriate system folder on your computer. However, some users—especially on shared university computers, for example—may not have the rights to write to system folders. If, when trying to install packages with this command, R returns an error saying that there are no rights to write into the system folder, you can run the function with the lib argument, specifying the folder where the packages should be installed. For example, install.packages("brms", lib = "C:/Users/example_user/Documents/Rpackages").

11 This function should install the brms package and all the software that it depends on. However, some users may need to also install a C++ toolchain. Detailed instructions for Mac and Linux users can be found at the official Stan documentation website (https://github.com/stan-dev/rstan/wiki/Installing-RStan-on-Mac-or-Linux#toolchain). Windows users will find equivalent instructions at https://github.com/stan-dev/rstan/wiki/Installing-RStan-on-Windows#toolchain.

12 To install this package, call install.packages("tidyverse").

13 Use R Studio’s “Files” panel to navigate to the folder on your computer that contains the R script and data file. Then click “More” -> “Set as Working Directory”.

14 Many readers might be more familiar with the equivalent “error-centric” representation of this model: $Y_{ij}=\mu_{ij}+\varepsilon_{ij}$, where the “errors” are normally distributed $\varepsilon_{ij}∼N(0,\sigma^{2})$.

15 For example, in a traditional ANOVA, if a participant provided a response in the first time point but not the second, that participant’s data would be discarded. In a multilevel model, the participant’s single observation can be used to inform the group’s estimate at the first time point. Additionally, the participant will have an estimated effect of the pre-post difference, equal to the group mean effect.

16 Notice that the $N(\beta_{0},\tau_{0})$ is a prior distribution for the person-level intercepts, whose parameters are themselves estimated from the data (but are also assigned “hyper”priors). For this reason, the person-specific intercepts are sometimes called empirical Bayes estimates.

17 If you installed R packages to a custom location, you also need to instruct the library() function to use the custom location (for example, library(brms, lib = "C:/Users/example_user/Documents/Rpackages")).

18 These priors are non-informative and only exist to help the underlying MCMC algorithms. For most purposes, they can be ignored.

19 For this tutorial, we ignore that brms specifies the intercept slightly differently. See ?set_prior for details.

20 More precisely, we approximate this from the MCMC samples by taking the proportion of samples from this parameter’s posterior distribution that are greater than zero.

21 Full source code for this reproducible manuscript, including all plots, is found at https://doi.org/10.5281/zenodo.1209814.

## Appendix

### Code for Figure 7

library(bayesplot) library(papaja) library(gridExtra) color_scheme_set("gray") fixef <- posterior_samples(fit_1, "b") ppars <- mcmc_areas(fixef, adjust = 1, prob = .95) +  labs(x = "Parameter value") +  theme_apa(base_size = 9) X <- list(intervention = setNames(0:1, c("Control", "Treatment"))) me <- marginal_effects(fit_1,             effects = "time:intervention",             int_conditions = X,             method = "fitted") pfits <- plot(me, plot = F)[[1]] +  scale_color_brewer(palette = "Set1") +  scale_fill_brewer(palette = "Set1") +  coord_cartesian(ylim = c(2, 5)) +  scale_x_continuous("Time", breaks = 0:1) +  labs(y="Fitted motivation") +  theme_apa(base_size = 9) +  theme(legend.position = "none",      axis.line = element_line()) grid.arrange(ppars, pfits, nrow=1)

### Code for Figure 10

pp_check(fit_1, nsamples = 100)

### Ordinal logistic model

In the main manuscript, we describe how to model the data assuming Gaussian outcomes. Another, perhaps more appropriate model would be to treat the outcomes (ratings) as discrete 1–5 ratings (Bürkner & Vuorre, 2018)[Bibr CIT0008]. In the main text, we avoided this model because it is less familiar and more complicated to interpret, but present the code here for interested readers. Notice that the model is fit to raw responses, and not averaged across rating items. Therefore we also add varying intercepts for items.

fit_2 <- brm(value ∼ time*intervention + (time|ID) + (1|item),         family = cumulative(link="logit"), cores = 4, data = d)

summary(fit_2)

pp_check(fit_2, nsamples = 100)
